# Crystal structure of μ-cyanido-1:2κ^2^ *N*:*C*-dicyanido-1κ*C*,2κ*C*-bis­(quinolin-8-amine-1κ^2^ *N*,*N*′)-2-silver(I)-1-silver(II): rare occurrence of a mixed-valence Ag^I,II^ compound

**DOI:** 10.1107/S2056989015009664

**Published:** 2015-05-23

**Authors:** Zouaoui Setifi, Sylvain Bernès, Olivier Pérez, Fatima Setifi, Djamil-Azzeddine Rouag

**Affiliations:** a Laboratoire de Chimie, Ingénierie Moléculaire et Nanostructures (LCIMN), Université Ferhat Abbas Sétif 1, Sétif 19000, Algeria; b Unité de Recherche de Chimie de l’Environnement et Moléculaire Structurale (CHEMS), Université Constantine 1, Constantine 25000, Algeria; c Instituto de Física, Benemérita Universidad Autónoma de Puebla, Av. San Claudio y 18 Sur, 72570 Puebla, Pue., Mexico; d Laboratoire CRISMAT, UMR 6508 CNRS, ENSICAEN, 6 Boulevard du Maréchal Juin, 14050 Caen Cedex 04, France

**Keywords:** crystal structure, silver, mixed valency, quinoline

## Abstract

The title dinuclear complex, [Ag_2_(CN)_3_(C_9_H_8_N_2_)_2_], may be considered as an Ag^II^ compound with the corresponding metal site coordinated by two bidentate quinolin-8-amine mol­ecules, one cyanide group and one dicyanidoargentate(I) anion, [Ag(CN)_2_]^−^. Since this latter ligand contains an Ag^I^ atom, the complex should be a class 1 or class 2 mixed-valence compound, according to the Robin–Day classification. The Ag^II^ atom is six-coordinated in a highly distorted octa­hedral geometry, while the Ag^I^ atom displays the expected linear geometry. In the crystal, the amino groups of the quinolin-8-amine ligands form N—H⋯N hydrogen bonds with the N atoms of the non-bridging cyanide ligands, forming a two-dimensional network parallel to (102). The terminal cyanide ligands are not engaged in polymeric bonds and the title compound is an authentic mol­ecular complex. The title mol­ecule is thus a rare example of a stable Ag^I,II^ complex, and the first mixed-valence Ag^I,II^ mol­ecular complex characterized by X-ray diffraction.

## Abstract 2

The title compound, [Ag_2_(CN)_3_(C_9_H_8_N_2_)_2_], is a mixed-valence disilver mol­ecular complex. The Ag^+^ ion has the expected linear coordination geometry, while the Ag^2+^ centre is six-coordinated with a distorted [AgN_5_C] octa­hedral geometry. This compound belongs to class 1 or class 2 complexes in the Robin–Day classification.

## Chemical context   

The coordination chemistry of silver is clearly dominated by Ag^I^ complexes. The oxidation state Ag^II^, with a paramagnetic 4*d* ^9^ electronic configuration, is however present in inorganic species like AgF_2_, a compound which readily decomposes in water, and is even able to oxidize SiCl_4_ (Grochala & Mazej, 2015 ▸). Ag^II^ is also stable in bimetallic perfluorinated compounds Ag^II^ *M* ^IV^F_6_, with *M* = Pt, Pd, Ti, Rh, Sn and Pb. In these solids, the Ag^II^ sites are bonded to six F atoms, in an octa­hedral coordination geometry distorted by the Jahn–Teller effect. In contrast, AgO, precipitated from Ag in presence of K_2_S_2_O_8_ in a basic medium, is a diamagnetic mixed-valence Ag^I,III^ oxide, rather than a Ag^II^ compound (Housecroft & Sharpe, 2012 ▸). Some actual Ag^II^ coordination complexes may be formed in solution, for example [Ag(bpy)_2_]^2+^, which follows the Curie law with a magnetic moment close to the spin-only value expected for a *d* ^9^ system (Kandaiah *et al.*, 2012 ▸).

Recently, polynitrile and cyanido­metallate anions have received considerable attention because of their importance in both coordination chemistry and in mol­ecular materials chemistry (Atmani *et al.*, 2008 ▸; Benmansour *et al.*, 2008 ▸, 2009 ▸, 2012 ▸; Setifi *et al.*, 2013 ▸; Setifi, Lehchili *et al.*, 2014 ▸; Setifi, Charles *et al.*, 2014 ▸). In view of the possible roles of these versatile anionic ligands, we have been inter­ested in using them in combination with other chelating or bridging neutral co-ligands to explore their structural and electronic charac­teristics in the extensive field of mol­ecular materials exhib­iting the spin-crossover (SCO) phenomenon (Dupouy *et al.*, 2008 ▸, 2009 ▸; Setifi *et al.*, 2009 ▸; Setifi, Charles *et al.*, 2014 ▸; Setifi, Milin *et al.*, 2014 ▸). During the course of attempts to prepare such complexes, using the di­cyanido­argentate(I) anion, we isolated the title compound, whose structure is described here.
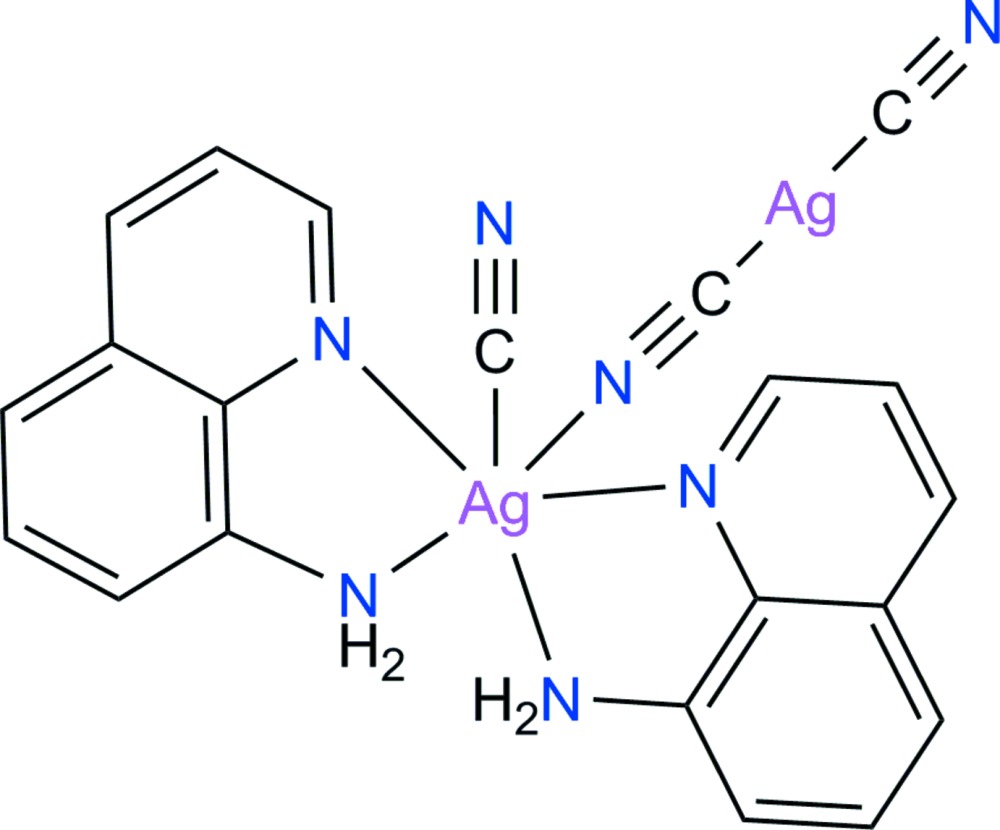


## Structural commentary   

**Figure 1:**
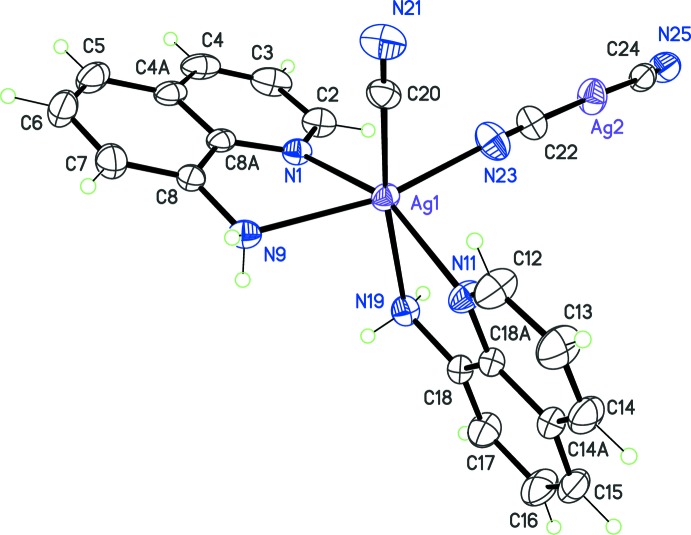
The mol­ecular structure of the title complex, with displacement ellipsoids drawn at the 30% probability level.

The title complex (Fig. 1 ▸) is a binuclear silver compound placed in a general position, in which metallic sites present contrasting coordination environments. Ag1 is six-coordinated by two quinolin-8-amine bidentate ligands, one terminal cyanide ligand, and one bridging cyanide ligand. The quinoline ring system N1–C8 is slightly twisted, with a r.m.s. deviation of 0.04 Å, while the other, N11–C18, may be considered as planar (rms deviation: 0.01 Å). Quinoline ligands are arranged *cis* in the octa­hedral coordination polyhedron, and their mean planes make a dihedral angle of 58.71 (5)°. The amino groups bonded to C8 and C18 are *trans* to the cyanide ligands. The octa­hedral geometry around Ag1 is distorted, mainly because of bite angles for quinoline ligands, N1—Ag1—N9 = 69.59 (7)° and N11—Ag1—N19 = 71.29 (7)°. The coordination of the terminal cyanide ligand, C20 N21 is through the C atom, as determined from the structure refinement (see *Refinement* section). This orientation seems to be favored by the availability of atom N21 as an acceptor for hydrogen bonding with symmetry-related mol­ecules in the crystal (Table 1 ▸).

Metal site Ag2 has a linear coordination with two cyanide ligands. Both ligands are coordinated through their C atoms (C22 and C24), and the coordination angle C22—Ag2—C24 = 176.05 (11)°, close to the ideal angle of 180° expected for an *sp* hybridization of the metal. Site Ag2 may thus be confidently assigned to a Ag^I^ coordination site, and charge balance for the complex should then set the oxidation state for the octa­hedral metal as Ag^II^, with a formal hybridization *sp* ^3^ *d* ^2^. The title complex is a mixed-valence compound, with valences localized on a single site. According to the Robin–Day classification (Day *et al.*, 2008 ▸), this compound should thus be a class 1 or class 2 mixed-valence compound. The deep-red color of the crystals should be the result of the π^*^←4*d*(Ag) metal-to-ligand charge transfer, rather than a consequence of an inter­valence charge transfer of a class 2 complex. Indeed, porphyrinato–Ag^II^ compounds are generally purple or red compounds (*e.g*. Xu *et al.*, 2007 ▸).

Cyanide ligand C22 N23 bridges metal sites Ag1 and Ag2, with oxidation states II and I respectively. The best structure refinement shows that this ligand is not disordered: the C atom is bonded to Ag^+^, and the N atom to the Ag^II^ atom. This orientation observed for the bridge is consistent with the Pearson’s HSAB principle (Pearson, 2005 ▸). The cyanide Lewis base is considered as a soft ligand, which preferentially forms covalent bonds with soft Lewis acid, like Ag^+^. However, the heteronuclear nature of this ligand induces an asymmetric character for the softness: based on the absolute electronegativity criterion, the C side of the cyanide ligand is expected to be softer than the N side. On the other hand, regarding the acid component of the coordination bonds, Ag^+^ is expected to be softer than Ag^2+^, due to the charge difference, which makes Ag^+^ more polarizable than Ag^2+^. The most stable acid–base inter­actions for the bridging mode of ligand C22 N23 is thus Ag^+^—C N—Ag^2+^, as observed in the X-ray-based structure refinement. From the reactivity point of view, the di­cyanido­argentate(I) anion, [Ag(CN)_2_]^−^, used as starting material, preserves the κC coordination mode for the cyanide groups in the product. This anion thus acts as a ligand to the oxidized Ag^II^ atom formed during the reaction. The same κC coordination is observed for the terminal cyanide group bonded to Ag^2+^, indicating that this fragment [Ag(CN)]^+^ is also produced from di­cyanido­argentate, probably prior to amino­quinoline coordination.

## Supra­molecular features   

**Figure 2:**
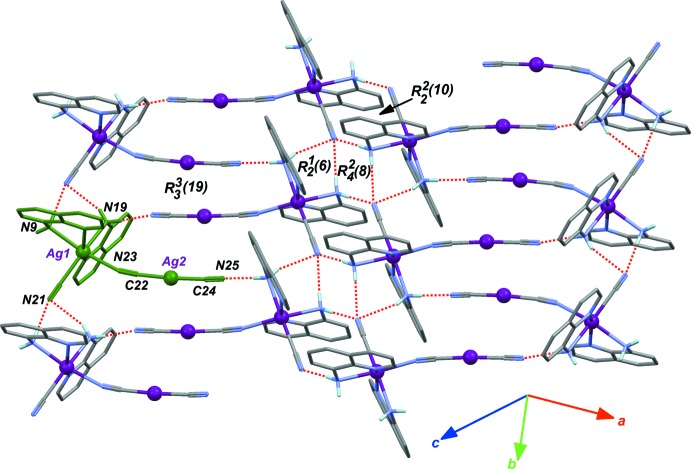
Part of the crystal structure of the title complex, emphasizing the N—H⋯N hydrogen bonds (dashed red lines) forming *R* rings. The green mol­ecule corresponds to the asymmetric unit.

As described in the previous section, both terminal cyanide ligands are bonded to Ag1 and Ag2 as κC ligands, allowing the N terminus to act as acceptor sites for hydrogen bonding (Ramabhadran *et al.*, 2014 ▸). Amino groups of amino­quinoline ligands are the donors for these contacts (Table 1 ▸), forming a two-dimensional supra­molecular network parallel to (102) (Fig. 2 ▸). Mol­ecules are aggregated through a centrosymmetric 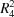
(8) ring, where the donor group is the terminal cyanide C20/N21 bonded to Ag1. The same cyanide ligand is engaged in 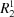
(6) rings, where donors are from two different amino groups. This basic pattern of fused rings propagates in the [010] direction, *via* larger 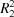
(10) rings. Finally, these rows of mol­ecules are connected in the crystal *via* the long arms Ag2—C24 N25, which take part in large 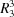
(19) rings. The shortest metal⋯metal distance is observed in these rings involving Ag^+^ ions: Ag2⋯Ag2^i^ = 3.9680 (3) Å [symmetry code (i): −*x* + 2, *y* + 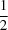, −*z* + 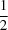].

Although the resulting supra­molecular structure is compact, hydrogen bonds, with H⋯N contacts in the range 2.19 (3)–2.48 (3) Å, should be considered as inter­actions of moderate strength. The crystallized compound is an authentic mol­ecular complex, in which the terminal cyanide ligands are not engaged in polymeric bonds.

## Database survey   

Complexes characterized by X-ray diffraction which include at least one Ag^2+^ ion are much less common than Ag^+^ complexes. An estimation using the field ‘NAME = silver(II)’ or ‘NAME = silver(I)’ in the current release of the CSD (version 5.36 with all updates; Groom & Allen, 2014 ▸), affords 63 and more than 8000 hits, respectively. Within Ag^I^ complexes, the occurrence of the di­cyanido­argentate ion is significant. It has been used not only as a counter-ion (*e.g*. Stork *et al.*, 2005 ▸) but also as a ligand for numerous transition-metal ions, including Ag^+^ (Lin *et al.*, 2005 ▸).

For non-polymeric compounds, the most common coordin­ation for Ag^2+^ is the square-planar [AgN_4_] arrangement, found in porphyrin derivatives and tetra-aza cyclic ligands (*e.g*. Xu *et al.*, 2007 ▸). However, a few cases of six-coordinate Ag^2+^ species have been characterized, with *N*-donor ligands (Clark *et al.*, 2009 ▸) and *S*-donor ligands (Shaw *et al.*, 2006 ▸). Compounds with both Ag^+^ and Ag^2+^ ions which have been X-ray characterized seem to be very scarce. A 1D polymeric mixed-valent Ag^I^/Ag^II^ polymer was obtained by reacting AgNO_3_, Na_2_S_2_O_8_ and pyrazine in a CH_3_CN/H_2_O mixture, and the presence of Ag^2+^ was confirmed by ESR (Sun *et al.*, 2010 ▸). The two other cases retrieved from the CSD are ionic compounds, in which tetra­aza­cyclo­tetra­decane derivatives coordinate the Ag^2+^ ion in a square-planar geometry, while the Ag^+^ ion is present in the anionic polymeric part (Wang & Mak, 2001 ▸) or in an anionic cluster (Wang *et al.*, 2002 ▸). The title complex is, as far we can see, the first non-polymeric and non-ionic mixed-valence Ag^I,II^ compound characterized by X-ray diffraction.

## Synthesis and crystallization   

The title compound was obtained under solvothermal conditions from a mixture of iron(II) sulfate hepta­hydrate (28 mg, 0.1 mmol), quinolin-8-amine (30 mg, 0.2 mmol) and potassium di­cyanido­argentate (40 mg, 0.2 mmol) in water–ethanol (4:1 *v*/*v*, 20 ml). The mixture was transferred to a Teflon-lined autoclave and heated at 423 K for 48 h. The autoclave was then allowed to cool to ambient temperature. Deep-red crystals of the title compound were collected by filtration, washed with water and dried in air (yield 30%).

## Refinement   

Crystal data, data collection and structure refinement details are summarized in Table 2 ▸. Special attention was paid to the accurate orientation for the three cyanide ligands in the asymmetric unit. For each C N group, two refinements were carried out with each possible orientation, and the best model was retained on the basis of *R*_1_ and *wR*_2_ factors, and ADP for the C and N sites. For example, *wR*_2_ for all data rises from 8.78% to *ca*. 9.30% if one cyanide ligand bonded to Ag2 is inverted. No evidence for disordered cyanido groups was detected in the difference maps. All C-bonded H atoms were placed in calculated positions and refined as riding atoms, with C—H bond lengths fixed to 0.93 Å. Amino H atoms bonded to N9 and N19 were found in a difference map and refined freely. For all H atoms, isotropic displacement parameters were calculated as *U*_iso_(H) = 1.2*U*_eq_(carrier atom).

## Supplementary Material

Crystal structure: contains datablock(s) I, global. DOI: 10.1107/S2056989015009664/lh5764sup1.cif

Structure factors: contains datablock(s) I. DOI: 10.1107/S2056989015009664/lh5764Isup2.hkl

CCDC reference: 1401857

Additional supporting information:  crystallographic information; 3D view; checkCIF report

## Figures and Tables

**Table 1:** Hydrogen-bond geometry (, )

*D*H*A*	*D*H	H*A*	*D* *A*	*D*H*A*
N9H9*A*N21^i^	0.79(3)	2.36(3)	3.143(4)	169(3)
N9H9*B*N21^ii^	0.85(3)	2.23(3)	3.075(3)	172(3)
N19H19*A*N21^ii^	0.77(3)	2.48(3)	3.205(4)	157(3)
N19H19*B*N25^iii^	0.90(3)	2.19(3)	3.087(4)	175(3)

Symmetry codes: (i) 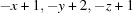; (ii) 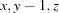; (iii) 
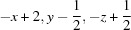
.

**Table 2:** Experimental details

Crystal data	
Chemical formula	[Ag_2_(CN)_3_(C_9_H_8_N_2_)_2_]
*M*_r_	582.15
Crystal system, space group	Monoclinic, *P*2_1_/*c*
Temperature (K)	293
*a*, *b*, *c* ()	13.5449(7), 6.9385(3), 22.3824(11)
()	94.767(2)
*V* (^3^)	2096.25(17)
*Z*	4
Radiation type	Mo *K*
(mm^1^)	1.89
Crystal size (mm)	0.27 0.23 0.18

Data collection	
Diffractometer	Bruker APEXII CCD
Absorption correction	Multi-scan (*SADABS*; Sheldrick, 2003 ▸)
*T*_min_, *T*_max_	0.615, 0.754
No. of measured, independent and observed [*I* > 2(*I*)] reflections	27103, 7113, 5226
*R*_int_	0.021
(sin /)_max_ (^1^)	0.750

Refinement	
*R*[*F* ^2^ > 2(*F* ^2^)], *wR*(*F* ^2^), *S*	0.034, 0.088, 1.02
No. of reflections	7113
No. of parameters	283
H-atom treatment	H atoms treated by a mixture of independent and constrained refinement
_max_, _min_ (e ^3^)	1.70, 0.56

Computer programs: *APEX2* and *SAINT* (Bruker, 2009 ▸), *SHELXS2014*/7 (Sheldrick, 2008 ▸), *SHELXL2014*/7 (Sheldrick, 2015 ▸) and *Mercury* (Macrae *et al.*, 2008 ▸).

## supplementary crystallographic information

### Crystal data

**Table:**

[Ag_2_(CN)_3_(C_9_H_8_N_2_)_2_]	*F*(000) = 1140
*M**_r_* = 582.15	*D*_x_ = 1.845 Mg m^−^^3^
Monoclinic, *P*2_1_/*c*	Mo *K*α radiation, λ = 0.71073 Å
*a* = 13.5449 (7) Å	Cell parameters from 9889 reflections
*b* = 6.9385 (3) Å	θ = 3.1–30.7°
*c* = 22.3824 (11) Å	µ = 1.89 mm^−^^1^
β = 94.767 (2)°	*T* = 293 K
*V* = 2096.25 (17) Å^3^	Prism, deep-red
*Z* = 4	0.27 × 0.23 × 0.18 mm

### Data collection

**Table:**

Bruker APEXII CCD diffractometer	5226 reflections with *I* > 2σ(*I*)
Radiation source: fine-focus sealed tube	*R*_int_ = 0.021
φ & ω scans	θ_max_ = 32.2°, θ_min_ = 4.2°
Absorption correction: multi-scan (*SADABS*; Sheldrick, 2003)	*h* = −20→17
*T*_min_ = 0.615, *T*_max_ = 0.754	*k* = −7→10
27103 measured reflections	*l* = −33→32
7113 independent reflections	

### Refinement

**Table:**

Refinement on *F*^2^	0 constraints
Least-squares matrix: full	Hydrogen site location: mixed
*R*[*F*^2^ > 2σ(*F*^2^)] = 0.034	H atoms treated by a mixture of independent and constrained refinement
*wR*(*F*^2^) = 0.088	*w* = 1/[σ^2^(*F*_o_^2^) + (0.043*P*)^2^ + 0.5603*P*] where *P* = (*F*_o_^2^ + 2*F*_c_^2^)/3
*S* = 1.02	(Δ/σ)_max_ = 0.001
7113 reflections	Δρ_max_ = 1.70 e Å^−^^3^
283 parameters	Δρ_min_ = −0.56 e Å^−^^3^
0 restraints	

### Fractional atomic coordinates and isotropic or equivalent isotropic displacement parameters (Å^2^)

**Table:**

	*x*	*y*	*z*	*U*_iso_*/*U*_eq_	
Ag1	0.66686 (2)	0.78323 (2)	0.41853 (2)	0.03575 (6)	
Ag2	0.97403 (2)	0.95903 (4)	0.28877 (2)	0.05800 (8)	
N1	0.58984 (15)	0.6944 (3)	0.32264 (9)	0.0372 (4)	
C2	0.6314 (2)	0.7024 (4)	0.27131 (12)	0.0487 (6)	
H2A	0.7001	0.7094	0.2722	0.058*	
C3	0.5762 (3)	0.7006 (4)	0.21569 (13)	0.0593 (8)	
H3A	0.6080	0.7002	0.1804	0.071*	
C4	0.4768 (3)	0.6994 (4)	0.21363 (13)	0.0584 (8)	
H4A	0.4397	0.7024	0.1768	0.070*	
C4A	0.4286 (2)	0.6937 (3)	0.26690 (12)	0.0470 (6)	
C5	0.3257 (2)	0.6965 (4)	0.26830 (17)	0.0631 (9)	
H5A	0.2852	0.7052	0.2327	0.076*	
C6	0.2850 (2)	0.6865 (4)	0.32116 (19)	0.0682 (9)	
H6A	0.2166	0.6928	0.3218	0.082*	
C7	0.3441 (2)	0.6670 (4)	0.37504 (15)	0.0552 (7)	
H7A	0.3142	0.6564	0.4109	0.066*	
C8	0.44478 (18)	0.6633 (3)	0.37580 (11)	0.0382 (5)	
C8A	0.48939 (18)	0.6834 (3)	0.32138 (11)	0.0360 (5)	
N9	0.50821 (17)	0.6373 (3)	0.42895 (10)	0.0400 (5)	
H9A	0.481 (2)	0.675 (4)	0.4567 (14)	0.048*	
H9B	0.525 (2)	0.519 (4)	0.4314 (13)	0.048*	
N11	0.74947 (17)	0.7366 (3)	0.51431 (9)	0.0445 (5)	
C12	0.7454 (2)	0.8572 (4)	0.55930 (13)	0.0617 (8)	
H12A	0.7000	0.9578	0.5554	0.074*	
C13	0.8057 (3)	0.8419 (5)	0.61242 (14)	0.0682 (9)	
H13A	0.8014	0.9322	0.6428	0.082*	
C14	0.8705 (2)	0.6948 (4)	0.61937 (14)	0.0597 (8)	
H14A	0.9111	0.6831	0.6548	0.072*	
C14A	0.87684 (19)	0.5588 (4)	0.57329 (12)	0.0448 (6)	
C15	0.9406 (2)	0.4006 (4)	0.57738 (15)	0.0590 (8)	
H15A	0.9829	0.3824	0.6118	0.071*	
C16	0.9414 (3)	0.2742 (4)	0.53187 (18)	0.0708 (10)	
H16A	0.9836	0.1684	0.5354	0.085*	
C17	0.8793 (2)	0.3000 (4)	0.47926 (14)	0.0560 (7)	
H17A	0.8815	0.2114	0.4482	0.067*	
C18	0.81619 (17)	0.4517 (3)	0.47276 (11)	0.0379 (5)	
C18A	0.81318 (16)	0.5856 (3)	0.52035 (10)	0.0362 (5)	
N19	0.75194 (17)	0.4838 (3)	0.41939 (10)	0.0404 (5)	
H19A	0.716 (2)	0.397 (4)	0.4210 (12)	0.048*	
H19B	0.788 (2)	0.478 (4)	0.3873 (14)	0.048*	
C20	0.6010 (2)	1.0782 (4)	0.44098 (11)	0.0431 (6)	
N21	0.5709 (2)	1.2156 (4)	0.45109 (12)	0.0620 (7)	
C22	0.8639 (2)	0.9377 (4)	0.34572 (14)	0.0525 (7)	
N23	0.8017 (2)	0.9129 (4)	0.37489 (12)	0.0607 (6)	
C24	1.0814 (2)	0.9611 (4)	0.23044 (13)	0.0497 (6)	
N25	1.1367 (2)	0.9559 (4)	0.19515 (12)	0.0620 (7)	

### Atomic displacement parameters (Å^2^)

**Table:**

	*U*^11^	*U*^22^	*U*^33^	*U*^12^	*U*^13^	*U*^23^
Ag1	0.03820 (10)	0.03710 (10)	0.03135 (10)	0.00115 (7)	−0.00073 (7)	−0.00062 (7)
Ag2	0.04638 (14)	0.07771 (16)	0.05085 (14)	−0.00800 (10)	0.00974 (10)	0.00022 (10)
N1	0.0449 (11)	0.0354 (10)	0.0311 (10)	0.0019 (8)	0.0019 (8)	−0.0006 (7)
C2	0.0625 (17)	0.0462 (14)	0.0379 (14)	0.0028 (12)	0.0082 (12)	−0.0016 (11)
C3	0.101 (3)	0.0448 (15)	0.0322 (14)	0.0020 (15)	0.0083 (15)	−0.0017 (11)
C4	0.096 (3)	0.0372 (14)	0.0383 (15)	0.0027 (14)	−0.0184 (15)	−0.0002 (11)
C4A	0.0630 (17)	0.0279 (11)	0.0464 (15)	0.0007 (10)	−0.0183 (13)	−0.0006 (10)
C5	0.0601 (19)	0.0490 (16)	0.074 (2)	0.0009 (13)	−0.0320 (17)	0.0012 (14)
C6	0.0419 (17)	0.0603 (19)	0.099 (3)	−0.0001 (13)	−0.0157 (18)	0.0021 (17)
C7	0.0449 (15)	0.0506 (14)	0.071 (2)	−0.0012 (12)	0.0079 (14)	0.0014 (14)
C8	0.0390 (13)	0.0296 (10)	0.0455 (14)	0.0001 (9)	−0.0001 (10)	0.0012 (9)
C8A	0.0457 (13)	0.0225 (9)	0.0383 (13)	0.0021 (8)	−0.0057 (10)	−0.0014 (8)
N9	0.0472 (12)	0.0381 (11)	0.0355 (11)	0.0048 (9)	0.0083 (9)	0.0019 (9)
N11	0.0491 (13)	0.0469 (11)	0.0359 (11)	0.0087 (9)	−0.0062 (9)	−0.0054 (9)
C12	0.074 (2)	0.0592 (17)	0.0497 (17)	0.0210 (15)	−0.0114 (15)	−0.0173 (14)
C13	0.089 (2)	0.0686 (19)	0.0438 (17)	0.0094 (18)	−0.0156 (16)	−0.0194 (15)
C14	0.068 (2)	0.0594 (17)	0.0474 (17)	0.0005 (14)	−0.0217 (14)	−0.0051 (13)
C14A	0.0402 (13)	0.0473 (14)	0.0450 (15)	−0.0041 (10)	−0.0074 (11)	0.0028 (11)
C15	0.0513 (17)	0.0565 (16)	0.065 (2)	0.0051 (13)	−0.0196 (14)	0.0039 (15)
C16	0.064 (2)	0.0536 (17)	0.091 (3)	0.0210 (14)	−0.0199 (18)	−0.0047 (17)
C17	0.0556 (17)	0.0464 (15)	0.0643 (19)	0.0098 (12)	−0.0041 (14)	−0.0121 (13)
C18	0.0334 (12)	0.0390 (12)	0.0408 (13)	−0.0025 (9)	0.0003 (10)	0.0000 (9)
C18A	0.0316 (11)	0.0386 (11)	0.0376 (13)	−0.0018 (9)	−0.0010 (9)	0.0017 (9)
N19	0.0422 (12)	0.0432 (11)	0.0359 (11)	−0.0028 (8)	0.0041 (9)	−0.0047 (9)
C20	0.0539 (15)	0.0388 (13)	0.0365 (13)	−0.0039 (11)	0.0025 (11)	0.0045 (10)
N21	0.0785 (19)	0.0514 (14)	0.0581 (16)	0.0082 (13)	0.0184 (14)	0.0060 (12)
C22	0.0526 (17)	0.0500 (15)	0.0553 (18)	−0.0087 (12)	0.0074 (14)	−0.0033 (12)
N23	0.0626 (16)	0.0563 (14)	0.0659 (17)	−0.0120 (12)	0.0221 (13)	−0.0064 (12)
C24	0.0423 (15)	0.0592 (16)	0.0470 (16)	0.0007 (12)	−0.0004 (13)	0.0082 (12)
N25	0.0548 (15)	0.0745 (17)	0.0581 (17)	0.0054 (12)	0.0119 (13)	0.0138 (12)

### Geometric parameters (Å, º)

**Table:**

Ag1—C20	2.305 (3)	N9—H9A	0.79 (3)
Ag1—N23	2.323 (3)	N9—H9B	0.85 (3)
Ag1—N11	2.357 (2)	N11—C12	1.314 (3)
Ag1—N19	2.375 (2)	N11—C18A	1.357 (3)
Ag1—N1	2.3878 (19)	C12—C13	1.389 (4)
Ag1—N9	2.404 (2)	C12—H12A	0.9300
Ag2—C24	2.033 (3)	C13—C14	1.347 (4)
Ag2—C22	2.047 (3)	C13—H13A	0.9300
N1—C2	1.322 (3)	C14—C14A	1.406 (4)
N1—C8A	1.361 (3)	C14—H14A	0.9300
C2—C3	1.398 (4)	C14A—C15	1.395 (4)
C2—H2A	0.9300	C14A—C18A	1.419 (3)
C3—C4	1.343 (5)	C15—C16	1.345 (5)
C3—H3A	0.9300	C15—H15A	0.9300
C4—C4A	1.407 (4)	C16—C17	1.400 (4)
C4—H4A	0.9300	C16—H16A	0.9300
C4A—C5	1.397 (4)	C17—C18	1.357 (3)
C4A—C8A	1.415 (3)	C17—H17A	0.9300
C5—C6	1.347 (5)	C18—C18A	1.417 (3)
C5—H5A	0.9300	C18—N19	1.436 (3)
C6—C7	1.397 (5)	N19—H19A	0.77 (3)
C6—H6A	0.9300	N19—H19B	0.90 (3)
C7—C8	1.363 (4)	C20—N21	1.069 (3)
C7—H7A	0.9300	C22—N23	1.121 (4)
C8—C8A	1.411 (4)	C24—N25	1.133 (4)
C8—N9	1.420 (3)		
			
C20—Ag1—N23	94.56 (9)	C8—N9—Ag1	110.41 (15)
C20—Ag1—N11	94.96 (8)	C8—N9—H9A	109 (2)
N23—Ag1—N11	96.04 (9)	Ag1—N9—H9A	114 (2)
C20—Ag1—N19	166.25 (8)	C8—N9—H9B	108.5 (19)
N23—Ag1—N19	86.81 (9)	Ag1—N9—H9B	100.1 (19)
N11—Ag1—N19	71.29 (7)	H9A—N9—H9B	114 (3)
C20—Ag1—N1	106.09 (8)	C12—N11—C18A	118.8 (2)
N23—Ag1—N1	91.27 (8)	C12—N11—Ag1	124.28 (18)
N11—Ag1—N1	157.10 (7)	C18A—N11—Ag1	116.52 (16)
N19—Ag1—N1	87.54 (7)	N11—C12—C13	123.3 (3)
C20—Ag1—N9	89.30 (9)	N11—C12—H12A	118.4
N23—Ag1—N9	160.78 (9)	C13—C12—H12A	118.4
N11—Ag1—N9	102.39 (8)	C14—C13—C12	119.2 (3)
N19—Ag1—N9	93.89 (8)	C14—C13—H13A	120.4
N1—Ag1—N9	69.59 (7)	C12—C13—H13A	120.4
C24—Ag2—C22	176.05 (11)	C13—C14—C14A	120.2 (3)
C2—N1—C8A	118.8 (2)	C13—C14—H14A	119.9
C2—N1—Ag1	125.78 (18)	C14A—C14—H14A	119.9
C8A—N1—Ag1	113.24 (15)	C15—C14A—C14	123.7 (3)
N1—C2—C3	122.6 (3)	C15—C14A—C18A	119.2 (2)
N1—C2—H2A	118.7	C14—C14A—C18A	117.1 (2)
C3—C2—H2A	118.7	C16—C15—C14A	120.5 (3)
C4—C3—C2	119.4 (3)	C16—C15—H15A	119.8
C4—C3—H3A	120.3	C14A—C15—H15A	119.8
C2—C3—H3A	120.3	C15—C16—C17	120.8 (3)
C3—C4—C4A	120.4 (3)	C15—C16—H16A	119.6
C3—C4—H4A	119.8	C17—C16—H16A	119.6
C4A—C4—H4A	119.8	C18—C17—C16	121.2 (3)
C5—C4A—C4	123.6 (3)	C18—C17—H17A	119.4
C5—C4A—C8A	119.4 (3)	C16—C17—H17A	119.4
C4—C4A—C8A	117.0 (3)	C17—C18—C18A	119.1 (2)
C6—C5—C4A	120.0 (3)	C17—C18—N19	122.9 (2)
C6—C5—H5A	120.0	C18A—C18—N19	118.1 (2)
C4A—C5—H5A	120.0	N11—C18A—C18	119.3 (2)
C5—C6—C7	121.1 (3)	N11—C18A—C14A	121.5 (2)
C5—C6—H6A	119.4	C18—C18A—C14A	119.2 (2)
C7—C6—H6A	119.4	C18—N19—Ag1	113.75 (15)
C8—C7—C6	120.8 (3)	C18—N19—H19A	100 (2)
C8—C7—H7A	119.6	Ag1—N19—H19A	112 (2)
C6—C7—H7A	119.6	C18—N19—H19B	109.0 (19)
C7—C8—C8A	119.2 (2)	Ag1—N19—H19B	108.9 (17)
C7—C8—N9	123.2 (3)	H19A—N19—H19B	113 (3)
C8A—C8—N9	117.6 (2)	N21—C20—Ag1	179.5 (3)
N1—C8A—C8	119.1 (2)	N23—C22—Ag2	174.7 (3)
N1—C8A—C4A	121.6 (2)	C22—N23—Ag1	163.6 (2)
C8—C8A—C4A	119.2 (2)	N25—C24—Ag2	175.2 (3)
			
C8A—N1—C2—C3	0.2 (3)	C18A—N11—C12—C13	2.2 (5)
Ag1—N1—C2—C3	−161.74 (19)	Ag1—N11—C12—C13	−170.2 (3)
N1—C2—C3—C4	3.0 (4)	N11—C12—C13—C14	−1.4 (6)
C2—C3—C4—C4A	−2.1 (4)	C12—C13—C14—C14A	0.1 (6)
C3—C4—C4A—C5	178.5 (3)	C13—C14—C14A—C15	−178.8 (3)
C3—C4—C4A—C8A	−1.9 (3)	C13—C14—C14A—C18A	0.3 (5)
C4—C4A—C5—C6	178.6 (3)	C14—C14A—C15—C16	178.7 (3)
C8A—C4A—C5—C6	−1.0 (4)	C18A—C14A—C15—C16	−0.4 (5)
C4A—C5—C6—C7	−2.1 (5)	C14A—C15—C16—C17	0.9 (6)
C5—C6—C7—C8	2.0 (5)	C15—C16—C17—C18	−0.6 (5)
C6—C7—C8—C8A	1.3 (4)	C16—C17—C18—C18A	−0.2 (4)
C6—C7—C8—N9	−177.8 (3)	C16—C17—C18—N19	179.5 (3)
C2—N1—C8A—C8	176.2 (2)	C12—N11—C18A—C18	178.3 (3)
Ag1—N1—C8A—C8	−19.6 (2)	Ag1—N11—C18A—C18	−8.7 (3)
C2—N1—C8A—C4A	−4.4 (3)	C12—N11—C18A—C14A	−1.7 (4)
Ag1—N1—C8A—C4A	159.72 (16)	Ag1—N11—C18A—C14A	171.31 (18)
C7—C8—C8A—N1	175.0 (2)	C17—C18—C18A—N11	−179.4 (3)
N9—C8—C8A—N1	−5.8 (3)	N19—C18—C18A—N11	0.9 (3)
C7—C8—C8A—C4A	−4.3 (3)	C17—C18—C18A—C14A	0.6 (4)
N9—C8—C8A—C4A	174.9 (2)	N19—C18—C18A—C14A	−179.2 (2)
C5—C4A—C8A—N1	−175.2 (2)	C15—C14A—C18A—N11	179.7 (3)
C4—C4A—C8A—N1	5.2 (3)	C14—C14A—C18A—N11	0.5 (4)
C5—C4A—C8A—C8	4.2 (3)	C15—C14A—C18A—C18	−0.3 (4)
C4—C4A—C8A—C8	−175.4 (2)	C14—C14A—C18A—C18	−179.5 (2)
C7—C8—N9—Ag1	−153.5 (2)	C17—C18—N19—Ag1	−172.6 (2)
C8A—C8—N9—Ag1	27.4 (2)	C18A—C18—N19—Ag1	7.2 (3)

### Hydrogen-bond geometry (Å, º)

**Table:**

*D*—H···*A*	*D*—H	H···*A*	*D*···*A*	*D*—H···*A*
N9—H9*A*···N21^i^	0.79 (3)	2.36 (3)	3.143 (4)	169 (3)
N9—H9*B*···N21^ii^	0.85 (3)	2.23 (3)	3.075 (3)	172 (3)
N19—H19*A*···N21^ii^	0.77 (3)	2.48 (3)	3.205 (4)	157 (3)
N19—H19*B*···N25^iii^	0.90 (3)	2.19 (3)	3.087 (4)	175 (3)

Symmetry codes: (i) −*x*+1, −*y*+2, −*z*+1; (ii) *x*, *y*−1, *z*; (iii) −*x*+2, *y*−1/2, −*z*+1/2.
